# Workplace Violence against Health Care Providers in Emergency Departments of Public Hospitals in Jordan: A Cross-Sectional Study

**DOI:** 10.3390/ijerph20043675

**Published:** 2023-02-19

**Authors:** Osama Al Khatib, Hana Taha, Lujain Al Omari, Mohammed Qussay Al-Sabbagh, Abdallah Al-Ani, Faysal Massad, Vanja Berggren

**Affiliations:** 1 Department of Family and Community Medicine, Faculty of Medicine, The University of Jordan, Amman 11942, Jordan; 2 Department of Pharmacology, Public Health and Clinical Skills, Faculty of Medicine, The Hashemite University, P.O. Box 330127, Zarqa 13133, Jordan; 3 Department of Neuroscience, Caring Science and Society, Karolinska Institutet, 141 52 Huddinge, Sweden; 4 Department of Neurology, University of Kansas Medical Center, Kansas City, KS 66160, USA; 5 Office of Scientific Affairs and Research, King Hussein Cancer Center, Amman 11941, Jordan

**Keywords:** emergency department, workplace violence, Jordan, physicians, nurses

## Abstract

Workplace violence (WPV) against healthcare providers is a serious problem that carries health, safety, and legal consequences. Healthcare providers working in emergency departments (ED) are more susceptible to WPV compared to other healthcare settings. This study aimed to assess the prevalence of physical and verbal violence against ED physicians and nurses in public hospitals in Amman, Jordan, and to explore the relationship between WPV and the socio-demographic characteristics of the participants. A quantitative descriptive cross-sectional study design was used to assess physical and verbal violence against ED physicians and nurses. A self-administered questionnaire was completed by 67 physicians and 96 nurses from three public hospitals in Amman. In the past year, 33% and 53% of the participants experienced physical and verbal violence, respectively. Compared to their female counterparts, males were more frequently physically (43.7% vs. 2.3%, *p*-value < 0.001) and verbally (61.3% vs. 29.5%, *p*-value < 0.001) abused. The main perpetrators of physical and verbal violence were the patients’ relatives. Out of 53 physical and 86 verbal abuse incidents, only 15 cases (10.8%) were followed up with legal persecution. In conclusion, there is a widespread occurrence of physical and verbal violence against ED physicians and nurses in the public sector hospitals in Jordan. A collaborative effort by all stakeholders should be instituted to ensure the safety of the physicians and nurses and to improve the quality of the healthcare provided.

## 1. Introduction

Workplace violence (WPV) against healthcare providers is a serious problem that has many health, safety, and legal consequences [1]. It disrupts healthcare settings all over the world [2]. The World Health Organization (WHO) defines the act of violence as “the intentional use of physical force, threatened or actual, against others, or against a group or community that either result in injury, death, psychological harm, or deprivation” [3]. Moreover, the available literature defines violence in healthcare as “any incidents where the staff are abused, threatened, or assaulted in circumstances relating to their work involving an explicit or implicit challenge to their safety, well-being, or health” [4]. The definition is expanded to include any behavior ranging from direct violence to nonphysical assault (e.g., verbal abuse) that may cause healthcare workers to believe that they are at risk [5]. The nursing literature defines three attributes associated with workplace violence including patient-healthcare worker relationship (i.e., dysfunctional relationships between healthcare workers and patients, patients’ relatives, or other healthcare workers which might aggravate acts of violence), power and powerlessness (i.e., organizational disputes which depowers healthcare workers), and behavior of the perpetrator (i.e., actions and biases of perpetrators which present themselves as a source of violence independent of institutional dynamics) [5].

WPV and its health consequences, as an occupational hazard and public health issue, have received much interest recently [6,7]. Hostile work environments are associated with many negative implications on the healthcare providers’ health and well-being [8,9], as well as on their patients, due to the deterioration of the quality of care [6]. Furthermore, violent situations can be costly and cause significant harm to the healthcare system as a whole [10,11]. In their systematic review of WPV against nurses in healthcare, Pariona-Cabrera and colleagues demonstrated that WPV impacts the physical and mental health of healthcare workers, reduces their quality of care, and increases their turnover intention [12]. All of the aforementioned factors reduce the organizational effectiveness of health institutions, thereby impacting patients’ well-being. The loss of trust between healthcare workers and their employers, emotional exhaustion, and depersonalization are some of the factors that make it harder for institutions to attract and retain healthcare workers and force many victims to resign [11,12,13]. Nonetheless, WPV is still underreported in many healthcare settings [14].

Doctors and nurses provide frontline patient care as they interact with patients and their families at health facilities [8,9]. Nursing has been identified as the profession with the highest risk of patient-related violence within the healthcare sector [15]. According to the literature, the frequent exposure of nurses to verbal and physical abuse in some hospitals has made it an accepted part of their job [14,15]. A systematic review of the factors related to aggression perpetrated against nurses showed that a significantly higher proportion of female nurses were subjected to verbal abuse, compared to male nurses who were more likely to be victims of physical abuse [16].

Since healthcare personnel working in the emergency department (ED) are considered the frontline providers of secondary healthcare services, they might be more susceptible to WPV compared to other hospital wards [17]. In a Turkish study assessing violence that ED physicians are exposed to, approximately 92% of respondents reported exposure to violence at least once in their professional lives, with verbal violence being the most common type of violence exposed and witnessed [18]. In another study conducted in the United States of America, 96% of ED physicians were subjected to verbal harassment, and 83% were verbally threatened [19].

In Jordan, WPV and aggression is a serious problem that is attracting more attention in public health research [20]. In one study that was conducted in 2012 to assess the prevalence of WPV among Jordanian nurses, the prevalence of verbal and physical abuse was 37% and 18%, respectively [21]. Another study that aimed to explore the incidence, characteristics, and contributing factors of WPV committed against nurses in hospital EDs, found that 76% of the included participants had encountered some type of violence, with verbal violence having an incidence of approximately five-folds than that of physical violence (63.9% vs. 11.9%, respectively) [20]. Furthermore, a study conducted by Al-Omari that assessed the prevalence of physical and verbal abuse towards nurses working in Jordanian public hospitals found that female nurses were less likely to report physical violence by half compared to male nurses [22]. They were, however, more vulnerable to verbal violence by one and a half folds compared to male nurses.

The social learning theory (SLT) stipulates that the acquisition and maintenance of human behaviors depend on interactions between personal factors, environmental factors, and the nature of the behavior itself [23]. Albert Bandura’s SLT model comprised several key constructs, including differential reinforcement (i.e., behaviors change as a result of positive or negative consequences), vicarious learning (i.e., learning through the observation of others), cognitive processes (i.e., ability to manipulate incentives to alter behavior), and reciprocal determinism (i.e., the reciprocal dynamic of effect between behaviors and personal and environmental factors) [24]. The SLT model was originally developed to explore criminality and deviant behaviors [25]; therefore, it could provide a conceptual framework against which WPV is carefully examined.

Safety in the workplace is one of the basic needs of healthcare providers, as it will increase their productivity and ability to manage patients. However, violence against healthcare workers in Jordan is increasing. Moreover, there is a dearth of studies conducted in Jordan that aims to assess WPV against ED physicians in particular. This study aims to assess the prevalence of physical and verbal violence among ED physicians and nurses in public hospitals in Amman, Jordan, and to explore the relationship between the WPV and the socio-demographic characteristics of the participants.

## 2. Methods

### 2.1. Study Design, Setting, and Sampling

A quantitative descriptive cross-sectional study design was adopted to assess physical and verbal WPV among ED physicians and nurses. This study was conducted in 2017 in Amman, the capital city and the biggest governorate in Jordan, which has a population of around 4 million [26]. The public sector consists of two major public programs: the Ministry of Health and the Royal Medical Services; The Ministry of Health runs 29 hospitals in 11 governorates, and the Royal Medical Services operate 10 hospitals [27]. There are 6952 physicians and 9309 registered nurses employed by the public health sectors [28]. The study sample consists of ED physicians and nurses recruited from three public hospitals in Amman: Prince Hamza Hospital with 397 available beds, Al Basheer Hospital which is the biggest public hospital in Jordan with 951 available beds, and Al Totangi Hospital with 138 available beds [29].

The convenience sample consisted of ED physicians and nurses working at the aforementioned hospitals who provided direct care to patients and who could comprehend the Arabic language. Physicians or nurses who had a working experience of less than one year in the ED or had no direct contact with patients were excluded from the study. All respondents were invited to participate in the study by their direct managers. The total number of physicians and nurses in the EDs of the selected hospitals who were asked to participate was 240 (85 physicians and 155 nurses) and the total number of the respondents who did partake in the study was 163 participants (67 physicians and 96 nurses), with a response rate of (68%).

### 2.2. Measuring Instrument

A self-administered questionnaire was used to collect the data on physical and verbal WPV against physicians and nurses. A modified version of the International Labour Office (ILO), International Council of Nurses (ICN), World Health Organization (WHO), and Public Services International (PSI) (2003) questionnaire for WPV was used for data collection. The questionnaire was developed by the WHO based on the feedback of nurses working in hospitals. Permission was obtained from the WHO to use and modify the questionnaire for data collection.

The questionnaire comprised 3 domains including sociodemographics (domain 1), physical WPV (domain 2), and verbal WPV (domain 3). The sociodemographic domain contained 13 items (11 direct questions, 2 follow-up questions) pertaining to age, gender (male vs. female), marital status (single, married, divorced, or widowed), occupation (physician vs. nurse), overall years of experience, years of experience in the emergency department, work-related details (i.e., working in shifts, working between 7 a.m. and 6 p.m.), presence of anxiety over WPV, procedures to report WPV, and whether healthcare workers were encouraged to report WPV. Examples of follow-up questions within this domain included: “*If there are procedures to report WPV, do you know how to use them?*”, and, “*If you are encouraged to report WPV, please specify by whom?*”.

The physical WPV domain comprised 16 items (2 direct questions, 14 follow-up questions) pertaining to having experienced physical WPV in the past 12 months, details of the experienced physical WPV (i.e., perpetrator, time, response), outcomes of physical WPV (i.e., injuries, need for medical treatment, and mental outcomes), concerned authorities’ reaction to physical WPV (e.g., type of investigative authority, consequences for the abuser, provided support, and satisfaction with the aforementioned), the reasoning for not reporting physical WPV (if applicable), and the frequency of witnessed physical WPV during the past 12 months.

The verbal WPV domain constituted 11 items exploring personal experiences of verbal WPV in the last 12 months and their associated details (e.g., perpetrator, frequency, and response), perceived mental outcomes of verbal WPV, concerned authorities’ reaction to physical WPV (e.g., investigative authority, consequences for the abuser, provided support, and satisfaction with the aforementioned), and reasons for not reporting verbal WPV. The original questionnaire titled, “Workplace Violence in the Health Sector—Country Case Study Research Instruments—Survey Questionnaire,” is provided on the WHO publications website [30]. The modified version of the questionnaire utilized in this study is provided as part of Supplementary Materials (File S1).

For the purpose of this study, the definitions of the different forms of violence were adapted from the WHO. However, associated examples were altered to fit a more conservative Middle Eastern community. Physical violence is defined as “The use of physical force against another person or group, that results in physical, sexual or psychological harm. This includes beating, kicking, slapping, stabbing, shooting, pushing, biting, pinching, among others”. Psychological violence (verbal violence) is defined as the “intentional use of power, including threat of physical force, against another person or group, that can result in harm to physical, mental, spiritual, moral or social development. This particularly include any kind of behavior that humiliates, degrades or otherwise indicates a lack of respect for the dignity and worth of an individual (e.g., yelling, screaming, shouting, threatening, insulting/name calling, blaming/scapegoating, and scolding among others)”.

The questionnaire was translated from English to Arabic by an individual who was proficient in both languages and then translated back from Arabic to English by another individual who was also proficient in both languages. Moreover, it was pilot tested with 20 nurses and 10 physicians. Modifications were performed to improve the understanding of some questions by participants. All participants in the pilot study were excluded from the main study sample. The instrument’s internal consistency was high (Cronbach’s alpha = 0.93).

### 2.3. Data Analysis

Data were entered and cleaned using Microsoft Excel. The study’s multitude of variables were coded per the nature of their outcome measure such as: level of satisfaction and frequency of mental complaints (5-item Likert-scale), perpetrator of WPV (categories ranging from patient, relatives of patient, staff, supervisor, among others), and experience of WPV (dichotomous yes/no scale). Interdependent relationships among the variables were measured by Pearson’s correlations, such as physical violence and verbal violence. Additional variables (age, gender, profession, and years of experience as an ED staff member) were analyzed by descriptive statistics. The data were also tested to determine if there was an association, if any, between workplace violence and the sociodemographic characteristics of the participants using the Chi-squared test. Data analysis was carried out using SPSS 20 (IBM Corp., Armonk, NY, USA). The level of statistical significance was set at *p*  <  0.05.

### 2.4. Ethical Considerations

The ethical approval of this study was issued by the Institutional Review Board (IRB) of Jordan’s Ministry of Health. The surveying tool was distributed together with a cover letter that included the aim of the study and information of the participants together with an informed consent. Confidentiality, voluntary, and full autonomy of participation were ensured. The participants had the right to withdraw from the study without justification at any time during the data collection.

## 3. Results

### 3.1. Participants’ Characteristics

Of the 163 ED healthcare workers who participated in this survey, 41.1% were physicians, while 58.9% were nurses. The majority of the recruited sample were males (73%) with a male-to-female ratio of approximately 3-to-1. Moreover, 77.3% were younger than 34 years of age, while 22.7% were older than 35 years. Among the participants, years of experience within a hospital setting ranged from one year to over 20 years. The mean number of years of experience for nurse participants in this study was 2.74 years. The majority of respondents had 10 or fewer years of experience (86.5%). Participants’ sociodemographic variables are presented in Table 1.

### 3.2. Incidence of Physical and Verbal Violence

Of the responding ED physician and nurses, 53 participants (33%) experienced some form of physical violence in the last year, and 46% had witnessed physical violence against their colleagues at least once. Nine out of the 53 physically attacked participants (17%) were injured, and seven of those injured required formal medical treatment. Factors associated with an increased risk of experiencing physical violence include gender and occupation (all *p*-value < 0.01). Only one female participant reported experiencing physical WPV during the past 12 months.

Additionally, 86 participants (53%) experienced verbal violence at least once in the past 12 months of the study. Of the factors associated with experiencing verbal violence, male healthcare workers were significantly more likely to experience verbal abuse (*p* < 0.001). On the other hand, age (*p*-value = 0.228), years of experience (*p*-value = 0.136), and occupation (*p*-value = 0.072) did not influence exposure to verbal violence (Table 2).

### 3.3. Perpetrators of Violence

Of the 33% (n = 53) of those who were physically attacked, 9.4% were attacked by the patients themselves and 90.6% were attacked by the patients’ relatives. Whereas, out of the 53% (n = 86) who were verbally abused, 62.8% were perpetrated by patients’ relatives, 27.9% by the patients themselves, 7.0% by other hospital staff members, and 2.3% by management/supervisors.

### 3.4. Responses to Physical and Verbal Violence

Of the 53 participants who were physically violated, the majority tried to stop the perpetrators by themselves (60.4%), defended themselves physically (15.1%), or pursued legal prosecution (11.3%) (Table 3). Conversely, of the 86 participants who were verbally abused, 33.7% told their abuser to stop, 22.1% took no action, 16.3% pretended it never happened, while only 10.5% pursued legal prosecution. Furthermore, out of all the possible sociodemographic characteristics, only occupation was significantly associated with response to experiencing physical violence (*p* = 0.031) (Table 4).

## 4. Discussion

The results of this study showed that violence, both physical and verbal, is disturbingly prevalent toward ED physicians and nurses. Fifty-three percent and 33% of ED physicians and nurses experienced verbal and physical abuse in the past 12 months, respectively. Furthermore, 17% of physical abuse were injury-related, of which, most of them required treatment.

Partridge et al. also obtained worrying results which demonstrated that 88% of ED workers, both medical and non-medical staff, had experienced verbal abuse and that 43% of them were victims of physical assault within the six months preceding data collection [31]. However, while our results show that physicians are significantly more likely to experience both verbal abuse and physical assault, results obtained in that study show that doctors are more prone to verbal abuse, while nurses are more likely to be physically harmed [31]. On the other hand, Gates et al. found no significant differences between doctors and nurses in the number of assaults they experienced [32]. The ED presents itself as a ripe environment for the aggravation of violence as it puts an additional cognitive load on individuals experiencing stressed decision making [33]. Patient pain and discomfort, family members’ stress, and fear of the unknown are some factors that may increase the emotional tension of caregivers or the patients themselves, thus predisposing them to project their senses of frustration and vulnerability in the form of acts of WPV [21].

Visitors also commonly contribute to WPV [34]. In fact, our results show that patients’ relatives were the main perpetrators of physical and verbal abuse. Other regional results also demonstrate that patients’ relatives are more likely to exhibit violence than the patients themselves [35,36,37]. AbuAlRub and Al Khawaldeh attribute the violent acts committed by visitors to the strong bonds that lie within family members in Arabic societies, which intensifies the stress brought about by the patient’s illness [36]. 

The aggression portrayed by visitors and patients is in concordance with Bandura’s SLT model [23]. According to Bandura, responses to different social situations are based on previous experiences [38]. However, since EDs are high-stress environments [10], holding situations to which visitors and patients may not be accustomed to, their responses will be unpredictable; some will become agitated and violent while others will demonstrate more self-control. SLT explains such variances by establishing that behaviors are learned [38]. Within a Jordanian ED context, violence is expected to strive due to the lack of serious consequences (enhances differential reinforcement), the prevalence of violence by others (enhances vicarious learning), and the assumed effectiveness of violence by perpetrators (enhances lack of cognitive regulation).

The SLT’s ability to anticipate and explain violence and aggressiveness has been well demonstrated throughout different populations, particularly children [39]. The SLT, irrespective of the model developer, assumes that all observed behavior is internalized, which is then promoted into action through a mix of reinforcement (e.g., lack of a negative consequence or presence of a positive outcome) and cognitive biases witnessed throughout the environment (e.g., hostile attribution biases). The SLT is powerful enough to detect the transition between deviant behavior, such as violence, and criminality [40].

It was satisfying to find that only 4.9% of the participants in our study were verbally abused by fellow staff members and supervisors. In the results that Rowe and Sherlock obtained, 27% of the participant nurses reported that they were verbally abused by other nurses and 22% were abused by doctors [41]. Our results show that verbal abuse is more tolerated than physical abuse; all victims of physical aggression made some form of a conscious response, while 22% of victims of verbal aggression took no action. Furthermore, while 60% of victims of physical aggression told the perpetrators to stop, only 33% of victims of verbal abuse did that. However, 3.8% of victims of physical abuse and 5.8% of victims of verbal abuse reported the incident to senior staff members. While these percentages are disappointingly low, they might also reflect the ED personnel’s lack of trust in their seniors and the hospital management, choosing instead to take matters into their own hands or to simply put the incident behind them. Hamdan and Abu Hamra reported that 40% of the abuse victims reported incidents to their supervisors or hospital management [37]. However, 39% indicated that incidences of violence are not worth reporting due to the following reasons: lack of trust in administrative action, poor or lack thereof of a reporting system, and lack of knowledge of whom to report to.

In our study, it was revealed that males are more common victims of both physical and verbal abuse than females. A similar pattern of abuse was expressed towards males over females, which is consistent with other regional studies [37,42]. While this might be attributed to prevalent cultural norms rejecting disrespect to females in these societies, Gates et al. suggest that this might be because females use non-aggressive techniques to de-escalate patients while males are often required to deal with aggressive patients [32]. Additionally, such a phenomenon might be attributed to the perceptions of society towards the role of females within the healthcare system. Female practitioners often face significant implicit and explicit gender-based biases [43]. Such can be documented within modern literature as early as the 1970s [44]. Due to a general preference for male practitioners, often described as more “competent”, “trustworthy”, and “experienced” among others [45,46,47], the role of male healthcare workers is significantly amplified within communities holding the aforementioned beliefs, which may associate them with an associated risk of higher responsibilities and complications, namely abuse.

Our findings also show that there is no significant association between years of experience or the age of the participants, and exposure to abuse. The literature holds no concordant information regarding the strength of these correlations. Gates et al. found that there was no significant difference in the frequency of violence for age [32]. Conversely, in a systematic review of violence against healthcare workers, Chakraborty et al. demonstrated that younger and less experienced workers were more likely to be attacked [48]. The latter can be attributed to two factors: firstly, senior healthcare personnel may have more experience in recognizing and dealing with violent patients [49], and secondly, hierarchal communities, such as that of Jordan, place immense values of respect for older individuals [37]. We also observed an interesting, yet insignificant, trend within our results: the prevalence of physical violence decreased across the increasing years of experience groups, while verbal violence increased. Such a phenomenon was documented among Pakistani ED workers [50]. We hypothesize that perpetrators of violence will avoid physical violence with senior workers due to the societal pressures of hierarchal societies but will attempt verbal abuse as a mediator of aggression as it is associated with a lesser likelihood of any form of concrete consequences or persecution.

The violence that ED workers are exposed to may carry unavoidable consequences. Hamdan and Abu Hamra found that ED workers have experienced mental health issues, such as hopelessness, disappointment, fear, anxiety, and guilt [37]. Moreover, verbally abused nurses may experience more stress and job dissatisfaction, miss work and provide suboptimal quality of care to patients [41]. This may lead to increased job turnover, which in turn necessitates the costly recruitment and training of new staff [41]. All this will undoubtedly decrease the quality of care received by patients. 

Policies and legislation targeting violent acts against the healthcare team should be developed and activated. Caruso et al. suggested the implementation of a two-level interventional system [51]. At the intra-institutional level, strategies should be developed to reduce institutional causes of violence (e.g., providing comfortable conditions to mitigate long waiting times precipitated by overcrowding). Moreover, institutions should provide a compact reporting system for WPV and support it with legislative action. Furthermore, the security infrastructure should be improved. Partridge et al. suggest employing ED security guards to ensure the safety of physicians and nurses [31]. Other measures include high-security cameras, alarm systems, and rapid access to police. Rowe and Sherlock suggest that managers should involve nurses in decisions regarding policies and procedures when possible [41]. At the extra-institutional level, the public perception of WPV should be targeted by awareness campaigns mediated by politicians, religious leaders, and the media.

The second level of intervention should target the personal characteristics and behaviors of healthcare workers. Healthcare teams, especially those who work in EDs, should be trained to properly deal with violence and be able to de-escalate a situation, and victims should be instructed to immediately report cases of abuse. It is also paramount to understand the perspective of patients and their visitors. The nurses enrolled in the study conducted by AlBashtawy and Aljezawy reported that waiting time, overcrowding, and the attitude displayed by the emergency staff all contributed to violence [52]. These issues also need to be carefully addressed. Nonetheless, it should be considered that many of the anti-WPV strategies described within the literature are not associated with any empirical metrics of effectiveness [48]; therefore, resource-scarce institutions or even countries might be hesitant to implement them without further testing.

## 5. Limitations

Our results should be interpreted with caution due to the following limitations. Firstly, the study adopts a cross-sectional design which is known to be associated with a variety of biases (e.g., selection bias) and produces a “snap-shot” of the targeted population. Secondly, the data collection tool might not have produced the most accurate of results due to its close-ended nature and association with recall and social desirability biases. Thirdly, the study was conducted in the public sector hospitals in Amman using a convenience sampling technique yielding a relatively small number of participants which may have impacted the generalizability of the results and power of our statistical analysis. 

Ideally, future studies should recruit a random sample of healthcare providers from the EDs in public and private hospitals. Furthermore, it should include an analysis of the formal reporting system of violent incidents by physicians and nurses.

## 6. Conclusions

The findings of this study revealed the widespread occurrence of physical and verbal violence against ED physicians and nurses in the public sector hospitals in Amman, Jordan. The results demonstrate that WPV poses a major problem to ED healthcare personnel. Therefore, a collaborative effort by all stakeholders should be instituted to ensure ED workers’ safety and well-being as a means to enhance the quality of provided healthcare. Moreover, further research should be conducted to explore the factors aggravating WPV against healthcare workers.

## Figures and Tables

**Table 1:** Sociodemographic characteristics of included participants.

Variable		n (%)
Gender	Male	119 (73.0)
	Female	44 (27.0)
Age	20–24	11 (6.7)
	25–29	51 (32.3)
	30–34	64 (39.3)
	35–39	28 (17.2)
	+40	9 (5.5)
Years of experience	1–5	74 (45.4)
	6–10	67 (41.1)
	11–15	15 (9.2)
	+16	7 (4.3)
Occupation	Physician	67 (41.1)
	Nurse	96 (58.9)

**Table 2:** Exposure to physical violence according to the characteristics of respondents (n = 163).

Variable		Exposure to Physical Violence			Exposure to Verbal Violence		
		No	Yes	*p*-Value	No	Yes	*p*-Value
Gender	Male	67 (56.3%)	52 (43.7%)	<0.001	46 (38.7%)	73 (61.3%)	<0.001
	Female	43 (97.7%)	1 (2.3%)		31 (70.5%)	13 (29.5%)	
Age	20–24	10 (90.9%)	1 (9.1%)	0.073	8 (72.7%)	3 (27.3%)	0.288
	25–29	28 (54.9%)	23 (45.1%)		22 (43.1%)	29 (56.9%)	
	30–34	44 (68.8%)	20 (31.2%)		27 (42.2%)	37 (57.8%)	
	35–39	20 (71.4%)	8 (28.6%)		16 (57.1%)	12 (42.9%)	
	+40	8 (88.9%)	1 (11.1%)		4 (44.4%)	5 (55.6%)	
Years of experience	1–5	46 (62.2%)	28 (37.8%)	0.082	36 (48.6%)	38 (51.4%)	0.592
	6–10	46 (68.7%)	21 (31.3%)		30 (44.8%)	37 (55.2%)	
	11–15	12 (80.0%)	3 (20.0%)		10 (66.7%)	5 (33.3%)	
	+16	6 (85.7%)	1 (14.3%)		1 (14.3%)	6 (85.7%)	
Occupation	Physician	32 (47.8%)	35 (52.2%)	<0.001	26 (38.8%)	41 (61.2%)	0.072
	Nurse	77 (80.2%)	19 (19.8%)		51 (53.1%)	45 (46.9%)	

**Table 3:** Participants of healthcare workers towards physical and verbal WPV.

Response	Physical Violence	Verbal Violence
Took no action	0 (0.0%)	19 (22.1%)
Tried to pretend it never happened	5 (9.4%)	14 (16.3%)
Told the person to stop	32 (60.4%)	29 (33.7%)
Tried to defend oneself physically	8 (15.1%)	7 (8.1%)
Reported it to a senior staff member	2 (3.8%)	5 (5.8%)
Told a colleague	0 (0.0%)	1 (1.2%)
Completed an incident/accident form	0 (0.0%)	2 (2.3%)
Pursued prosecution	6 (11.3%)	9 (10.5%)
Total	53 (100%)	86 (100%)

**Table 4:** Factors affecting propensity to take action against workplace violence.

Variable		Actions against Verbal Violence			Actions against Physical Violence		
		Didn’t Take Action	Took Action	*p*-Value	Didn’t Take Action	Took Action	*p*-Value
Gender	Male	29 (39.7%)	44 (60.3%)	0.541	5 (9.6%)	47 (90.4%)	N/A
	Female	4 (30.8%)	9 (69.2%)		0 (0.0%)	1 (100.0%)	
Occupation	Physician	17 (41.5%)	24 (58.5%)	0.574	1 (2.9%)	33 (97.1%)	0.031
	Nurse	16 (35.6%)	29 (64.4%)		4 (21.1%)	15 (78.9%)	
Experience	<10 years	28 (37.3%)	47 (62.7%)	0.605	4 (8.2%)	45 (91.8%)	N/A
	>10 years	5 (45.5%)	6 (54.5%)		1 (25.0%)	3 (75.0)	
Age	<30 years	12 (37.5%)	20 (62.5%)	0.898	1 (4.2%)	23 (95.8%)	0.362
	>30 years	21 (38.9%)	33 (61.1%)		4 (13.8%)	25 (86.2%)	

## Data Availability

The data that support the findings of this study are available from the first author, [O.A.K.], upon reasonable request.

## Supplementary Materials

The following supporting information can be downloaded at: https://www.mdpi.com/article/10.3390/ijerph20043675/s1, File S1: Questionnaire.
