# Supplementary material for: Workplace Violence against Health Care Providers in Emergency Departments of Public Hospitals in Jordan: A Cross-Sectional Study
Source: Int J Environ Res Public Health. 2023 Feb 19;20(4):3675. doi: 10.3390/ijerph20043675 (PMC9964576; doi:10.3390/ijerph20043675)
Supplement: Supplementary file 1 [file ijerph-20-03675-s001.zip › ijerph-2215212-supplementary.pdf]

WORKPLACE VIOLENCE IN THE HEALTH SECTOR COUNTRY CASE STUDIES RESEARCH INSTRUMENTS

A MODIFIED SURVEY

Personal and workplace data

1. What is your age
   a. 20 to 24
   b. 25 to 29
   c. 30 to 34
   d. 35 to 39
   e. 40 to 44
   f. 45 to 49
   g. 50 to 54
   h. 55 to 59
   i. 60+
2. Gender
   a. Female
   b. Male
3. Marital status
   a. Single
   b. Married
   c. Divorced
   d. Widowed
4. Which category best describes your present professional group
   a. Physician
   b. Nurse
5. Years of experience (Overall)
   a. Under 1 year
   b. 1 to 5
   c. 6 to 10
   d. 11 to 15
   e. 16 to 20
   f. Over 20
6. Years of experience in an emergency department
   a. Under 1 year
   b. 1 to 5
   c. 6 to 10
   d. 11 to 15
   e. 16 to 20
   f. Over 20
7. Do you work in shifts
   a. Yes
   b. No
8. Do you work anytime between 18h00 (6 PM) and 07h00 (7 AM)?
   a. Yes
   b. No
9. How worried are you about violence in your current workplace?
   a. 1 = not worried at all
   b. 2
   c. 3

    d. 4
    e. 5 = very worried
10. Are there procedures for the reporting of violence in your workplace
    a. Yes
    b. No
        i. Follow-up: if, yes, do you know how to use them
            1. Yes
            2. No
11. Is there encouragement to report workplace violence
    a. Yes
    b. No
        i. Follow-up: if, yes, by whom
            1. Manager
            2. Union
            3. Family/friends
            4. Colleagues
            5. Others

Physical workplace violence

1. In the last 12 months, have you been physically attacked in your workplace?
    a. Yes
    b. No
        i. Follow-up: if, yes, who attacked you
            1. Patient
            2. Staff
            3. External worker
            4. Relative of patient
            5. Manager
            6. General public
            7. Other
        ii. Follow-up: at which time did it happen
        iii. Follow-up: how did you respond
            1. Took no action
            2. Tried to pretend it never happened
            3. Told the person to stop
            4. Tried to defend oneself physically
            5. Reported it to a senior staff member
            6. Told a colleague
            7. Completed an incident/accident form
            8. Pursued prosecution
        iv. Follow-up: Do you think the incident could have prevented
            1. Yes
            2. No
        v. Follow-up: Were you injured as a result of the violent incident?
            1. Yes
            2. No
                a. Follow-up: If, yes, did you require formal treatment
                    i. Yes
                    ii. No

vi. Follow-up: Please indicate how bothered you have been by these experiences since you were attacked (On a scale of Not at all, A little bit, moderately, quite a bit, extremely
    1. Repeated, disturbing memories, thoughts, or images of the attack?
    2. Avoiding thinking about or talking about the attack or avoiding having feelings related to it?
    3. Being "super-alert" or watchful and on guard?
    4. Feeling like everything you did was an effort?

vii. Was any action taken to investigate the causes of the incident
    1. Yes
    2. No
    3. I don’t know
        a. Follow-up: if, yes, by whom
            i. Manager
            ii. Union
            iii. Association
            iv. Community
            v. Police
            vi. Other
        b. Follow-up: What were the consequences for the attacker?
            i. None
            ii. Verbal warming
            iii. Care discontinued
            iv. Reported to police
            v. Aggressor prosecuted
            vi. I don’t know
            vii. Other

viii. Did your employer or supervisor offer to provide you with:
    1. Counselling
    2. Opportunity to speak about/report it
    3. Other support?

ix. How satisfied are you with the manner in which the incident was handled?
    1. 1 = Very dissatisfied
    2. 2
    3. 3
    4. 4
    5. 5 = Very satisfied

x. If you did not report or tell about the incident to others, why not?
    1. It was not important
    2. Felt ashamed
    3. Felt guilty
    4. Afraid of negative consequences
    5. Useless
    6. Don’t know who to report to

2. In the last 12 months, have you witnessed incidents of physical violence in your workplace
    a. Yes
    b. No
        i. Follow-up: if, yes, how often has this occurred in the last 12 months?
            1. Once
            2. 2 to 4 times
            3. 5 to 10 times

4. Several times a month
5. About once a week
6. Daily

Verbal abuse

1. In the last 12 months, have you been verbally abused in your workplace?
    a. Yes
    b. No
2. How often have you been verbally abused in the last 12 months?
    a. All the time
    b. Sometimes
    c. Once
3. Please think of the last time you were verbally abused in your place of work. Who verbally abused you?
    a. Patient
    b. Staff
    c. External worker
    d. Relative of patient
    e. Manager
    f. General public
    g. Other
4. How did you respond to the verbal abuse?
    a. Took no action
    b. Tried to pretend it never happened
    c. Told the person to stop
    d. Tried to defend oneself physically
    e. Reported it to a senior staff member
    f. Told a colleague
    g. Completed an incident/accident form
    h. Pursued prosecution
5. Please indicate how bothered you have been by these experiences since you were attacked (On a scale of Not at all, A little bit, moderately, quite a bit, extremely
    a. Repeated, disturbing memories, thoughts, or images of the attack?
    b. Avoiding thinking about or talking about the attack or avoiding having feelings related to it?
    c. Being "super-alert" or watchful and on guard?
    d. Feeling like everything you did was an effort?
6. Was any action taken to investigate the causes of the verbal abuse ?
    a. Yes
    b. No
    c. I don’t know
        i. Follow-up: if, yes, by whom
            1. Manager
            2. Union
            3. Association
            4. Community
            5. Police
            6. Other
        ii. Follow-up: What were the consequences for the attacker?
            1. None
            2. Verbal warming

3. Care discontinued
4. Reported to police
5. Aggressor prosecuted
6. I don’t know
7. Other

7. Did your employer or supervisor offer to provide you with:
   a. Counselling
   b. Opportunity to speak about/report it
   c. Other support?
8. How satisfied are you with the manner in which the incident was handled?
   a. 1 = Very dissatisfied
   b. 2
   c. 3
   d. 4
   e. 5 = Very satisfied
9. If you did not report or tell about the incident to others, why not?
   a. It was not important
   b. Felt ashamed
   c. Felt guilty
   d. Afraid of negative consequences
   e. Useless
   f. Don’t know who to report to
   g. Other

Modified version from: https://www.who.int/publications/m/item/workplace-violence-in-the-health-sector---country-case-study-research-instruments---survey-questionnaire
